# Enzymes and Metabolites in Carbohydrate Metabolism of Desiccation Tolerant Plants

**DOI:** 10.3390/proteomes4040040

**Published:** 2016-12-15

**Authors:** Qingwei Zhang, Xiaomin Song, Dorothea Bartels

**Affiliations:** Institute of Molecular Physiology and Biotechnology of Plants (IMBIO), University of Bonn, Kirschallee 1, 53115 Bonn, Germany; qzha@uni-bonn.de (Q.Z.); s6xisong@uni-bonn.de (X.S.)

**Keywords:** resurrection plants, sugar accumulation, enzymes catalyzing sugar metabolism, carbon distributions during dehydration, desiccation tolerance mechanism

## Abstract

Resurrection plants can tolerate extreme water loss. Substantial sugar accumulation is a phenomenon in resurrection plants during dehydration. Sugars have been identified as one important factor contributing to desiccation tolerance. Phylogenetic diversity of resurrection plants reflects the diversity of sugar metabolism in response to dehydration. Sugars, which accumulate during dehydration, have been shown to protect macromolecules and membranes and to scavenge reactive oxygen species. This review focuses on the performance of enzymes participating in sugar metabolism during dehydration stress. The relation between sugar metabolism and other biochemical activities is discussed and open questions as well as potential experimental approaches are proposed.

## 1. Introduction

Water availability is one of the most important ecological factors and evolutionary constraints that determine terrestrial life [1]. Excessive water loss is lethal to most animals and annual plant species. However, some animals, plants, and microbes can tolerate complete desiccation [2]. The ability to tolerate moderate to extreme desiccation and then to revive upon rehydration is termed ‘desiccation tolerance’ or ‘anhydrobiosis’. Desiccation tolerance is commonly found in reproductive organs of green plants (spores [3], seeds [4], and pollen [5]). For vegetative tissues, desiccation tolerance is common (though not universal) in bryophytes [6], rare in pteridophytes and angiosperms, and absent in gymnosperms [2,7,8]. Plants with vegetative desiccation tolerance are often termed ‘resurrection plants’.

Desiccation tolerance in resurrection plants is a multi-genic and multi-factorial phenomenon and the mechanism of desiccation tolerance is associated with preventing oxidative damage and maintaining native structures of macromolecules and membranes [9,10,11]. In plants, sugars function as substrates for intermediary metabolism and as signaling molecules, thus linking the carbon nutrient status with plant growth and development [12]. Sugar metabolism and sugar signaling also function in an intricate network linked with phytohormones and the production of reactive oxygen species (ROS) which also act as signaling molecules. The synergistic interaction of sugars (or sugar-like compounds) may form part of an integrated redox system, quenching ROS and contributing to stress tolerance, especially in tissues or organelles with plenty of soluble sugars [13]. Sugars have been shown to play an important role in the mechanism of plant desiccation tolerance [9,14]. Various studies on sugars in resurrection plants have been conducted, including identification and expression analysis of genes, activity analysis of enzymes, and biophysical investigations [15,16,17,18].

Although a correlation of sugar accumulation and desiccation tolerance is accepted, the mechanism by which sugars function is not really understood. It is not known how individual compounds such as sugars and protective proteins interact spatially and temporally. The integration of the individual components required for desiccation tolerance into a coherent functional framework is difficult. Therefore, this review focuses on sugar metabolism in desiccation tolerant plants. The review describes the diversity of sugars accumulating in plants during dehydration, the performance of enzymes in sugar metabolism and sugar metabolism networks revealed by proteomics and metabolomics. An attempt is made to present an integrated and systematic understanding of sugar metabolism in plant desiccation tolerance.

## 2. Accumulation of Sugars in Response to Dehydration

Sugar accumulation is one of the principal phenomena in resurrection plants during dehydration. The changes of sugar metabolism reported for resurrection plants are summarized in Table 1. A general observation is that high amounts of sucrose accumulate when resurrection plants encounter dehydration. However, there is also a species-specific diversity of sugar metabolism in resurrection plants and often the metabolic pathway leading to sucrose varies in desiccation tolerant species. In some species, unusual sugars accumulate: e.g., octulose in *Craterostigma plantagineum* or trehalose in *Myrothamnus flabellifolia* are abundant. In some resurrection species—e.g., *Xerophyta viscosa*—raffinose family oligosaccharides accumulate abundantly besides sucrose during dehydration.

## 3. Physiological Roles of Sugars Accumulating in Resurrection Plants

Sucrose plays an important role in adjusting cellular osmotic potential, stabilizing macromolecules, and stabilizing cellular structural components [8]. The hypothesis is that hydroxyl groups of sugars replace water molecules and together with proteins facilitate glass formation of the cytoplasm [29]. Recent in vitro experiments indicate that sucrose also has hydroxyl radical scavenging ability during Fenton reactions with Fe^2+^ and hydrogen peroxide in vitro, thus participating in non-enzymatic reactions with hydroxyl radicals, forming an integral part of plant antioxidant mechanisms and contributing to cellular ROS homeostasis [30].

The raffinose family oligosaccharides (RFOs), such as raffinose and stachyose are synthesized from sucrose by the subsequent addition of activated galactinol moieties [31]. Galactose is linked to myo-inositol in the activated galactinol molecule which participates in the synthesis of RFOs. The synthesis of RFOs in resurrection plants may explain the occurrence of myo-inositol and galactinol, the former is linked with galactose to form the latter which participates in the synthesis of RFOs. RFOs are generally characterised as compatible solutes and as part of stress tolerance mechanisms. In addition, they also function as carbon transport and storage molecules [32], in signal transduction [33], membrane trafficking [34], and mRNA export [35]. Additional evidence accumulates that galactinol and RFOs protect plant cells from oxidative damage caused by various types of stress conditions [36,37].

Several resurrection plants such as *Eragrostis nindensis* [21], *Selaginella lepidophylla* [20], and *M. flabellifolius* [38] contain high levels of trehalose, a non-reducing disaccharide. Trehalose has been considered as the main factor in the acquisition of desiccation tolerance in Selaginella species [39,40]. However, accumulation of trehalose per se does not seem to be sufficient, because higher trehalose levels exist in the desiccation sensitive *Selaginella moellendorffii* than in its desiccation tolerant relative *S. lepidophyllals* [20]. Also, other desiccation tolerant plants contain, if at all, only small amounts of trehalose [21]. This implies that high trehalose levels are not necessarily required for desiccation tolerance [40]. However, trehalose has the ability to stabilize biological membranes, liposomes, and proteins in the dry state by its direct interaction with lipids and proteins of membranes. The direct interaction lowers the phase transition temperature of membranes to prevent leakage during rehydration and to preserve the native structure of labile proteins in the dry state [41]. Recently, a new role for trehalose was elucidated in the induction of mammalian m-TOR independent autophagy pathways [42]. In the desiccation tolerant plant *Tripogon loliiformis*, trehalose treatment triggers autophagy in vitro and trehalose accumulation correlates with the presence of autophagosomes in dehydrated leaves [43]. Thus, Williams et al. (2015) postulated that some resurrection plants modulate trehalose metabolism to induce and maintain autophagy pathways to prevent senescence and programmed cell death [43].

The accumulation of octulose seems to be correlated with desiccation tolerance within the Linderniaceae family, as high amounts of octulose accumulate in desiccation tolerant species [26,44]. Upon dehydration, most octulose is converted to sucrose [25,26] and therefore octulose could represent a sugar which is metabolically inert and can be rapidly converted to sucrose without interfering with the primary metabolism. Zhang and Bartels (2016) demonstrated that octulose has hydroxyl scavenging abilities superior to other common sugars in *C. plantagineum* and probably participates in oxidative stress defense reactions [45].

## 4. Studies on Enzymes Responsible for Sugar Accumulation in Resurrection Plants

Sugar metabolism is determined by biosynthesis enzymes and catabolizing enzymes. It is important to understand the performance of these enzymes in the context of plant adaption to desiccation.

Sucrose is synthesized in the cytosol. The principal sucrose-biosynthesis route involves sucrose-phosphate synthase (SPS; E.C. 2.4.1.14) and sucrose-phosphate phosphatase (SPP; E.C. 3.1.3.24). The former catalyzes the synthesis of sucrose-6-phosphate and the latter yields free sucrose and inorganic phosphate [46]. In 1997, Ingram et al. reported the isolation of two distinct classes of cDNAs encoding SPS from *C. plantagineum* [47]. The increase in activity of SPS in the leaves of *C. plantagineum* coincides with major sucrose accumulation. Compared with large changes of the mRNA during dehydration, the SPS protein level changed only slightly during dehydration. Therefore, it is proposed that the increase in enzyme activity may not only be attributed to the increased level of SPS protein, but also the activation state of the enzyme. Sucrose synthase (SuS; *E.C.* 2.4.1.13) catalyzes a readily reversible reaction and may be involved in both the synthesis and cleavage of sucrose [48]. However, SuS is usually assigned a role in sucrose cleavage in sucrose-consuming tissues under most physiological conditions, supplying monosaccharides as precursors of structural and storage polysaccharide biosynthesis [49]. Kleines et al. (1999) characterized two classes of cDNA and genomic clones encoding SuS from *C. plantagineum* [50]. During dehydration, SuS proteins accumulated in leaves and roots, which suggests that SuS also participates in sucrose accumulation in *C. plantagineum*.

The supply of hexoses is an important step in regulating sugar biosynthesis during dehydration. By examining leaf hexokinase activity and in vivo metabolite levels during drying in desiccation-tolerant *Sporobolus stapfianus* and *X. viscosa*, Whittaker et al. (2001) found that leaf hexokinase activity was significantly induced when the relative water content (RWC) in *S. stapfianus* was reduced from 85% to 29% and from 89% to 55% in *X. viscosa* [22]. The increase of hexokinase activities correlated with sucrose accumulation in both species. The decline of hexose sugars and accumulation of sucrose in both species was not associated with a decline in acid and neutral invertase. In contrast to hexokinase, fructokinase activity did not change during dehydration [22]. Therefore, fructose-6-phosphate may be originated from glucose-6-phosphate through other pathways, e.g., the pentose phosphate pathway and glycolysis for supporting sucrose synthesis during dehydration. This hypothesis is supported by the study of Velasco et al. (1994) [51] in which mRNA and protein levels of glyceraldehyde-3-phosphate dehydrogenase (GAPDH) increased and GAPDH enzyme activity became three to four-fold higher during dehydration, suggesting that enhanced rates of glycolysis might produce fructose-6-phosphate for sucrose synthesis.

Trehalose is synthesized in two steps whereby the first enzyme, trehalose-6-phosphate synthase (TPS) produces the intermediate trehalose-6-phosphate, which is then dephosphorylated by the second enzyme trehalose-6-phosphate phosphatase (TPP) [52]. A plant cDNA, SlTPS1, was isolated from *S. lepidophylla* [53]. SlTPS1 mRNA is constitutively expressed in *S. lepidophylla*. Transformation of a *Saccharomyces cerevisiae tps1Δ* mutant disrupted in the ScTPS1 gene with *S. lepidophylla* SlTPS1 restored growth on fermentable sugars and the synthesis of trehalose at high levels. For the second step, two genes encoding trehalose-6-phosphate phosphatase (TPP) were isolated and were able to complement the yeast *tps2* mutant for growth at 38 °C, indicating that they encode active enzymes. In addition, the enzymatic activity was not higher compared to the TPP enzymes of *A. thaliana* [51]. The reason for a high trehalose level in *S. lepidophylla* might be that the SlTPS1 enzyme is constitutively processed, resulting in very high enzymatic activity [54]. Márquez-Escalante et al. (2006) suggested that the TPS protein complexes in *S. lepidophylla* are also necessary for efficient regulation of trehalose biosynthesis [55].

Several resurrection plants accumulate myo-inositol which is involved in the synthesis of raffinose family oligosaccharides. The synthesis of myo-inositol from glucose includes three steps: (1) glucose-6-phoshate formation catalyzed by hexokinase; (2) cyclization of glucose-6-phosphate to myo-inositol-1-phosphate, catalyzed by myo-inositol-1-phosphate synthase; (3) the release of free myo-inositol catalyzed by myo-inositol monophosphatase [56]. The second step is the first committed step in myo-inositol biosynthesis. The gene encoding myo-inositol-1-phosphate synthase in *X. viscosa* (XINO1) was characterized by Majee et al. [57]. The gene has been identified by complementation of a yeast inositol-auxotrophic strain. The XINO1 gene product is catalytically active in a broad range of lower temperatures (between 10–40 °C) in contrast to the rice gene. The myo-inositol level is reduced during dehydration in several resurrection plants (Table 1) and there is a negative correlation between RFOs accumulation and myo-inositol depletion in *X. viscosa* during water deficit [23]; the performance of myo-inositol monophosphatase needs to be investigated in response to dehydration.

Due to the frequent presence of RFOs in resurrection plants, related enzymes have been explored. Galactinol synthase is responsible for the first catalytic step in RFOs biosynthesis. The gene XvGolS encoding galactinol synthase was characterized in *X. viscosa* [23]. In leaf tissue exposed to water deficit, *XvGolS* transcript levels increased at 19% RWC, but GolS activity decreased when RFOs accumulated [23]. Similarly, activities of galactinol synthase, raffinose synthase, and stachyose synthase decreased strongly during dehydration in leaves of *C. plantagineum* [26]. The decrease of GolS activity correlated with a decline of leaf galactinol concentrations while raffinose synthase and stachyose synthase were inversely correlated with the accumulation of raffinose and stachyose [26]. One possible explanation for the increased raffinose and stachyose levels in leaves could be that RFOs are transported from roots to leaves, as RFOs also represent sugar transport forms [32].

## 5. Sugar Metabolism during Dehydration Studied by Proteomics

Even though correlations have been established between dehydration stress and the activities of sugar metabolizing enzymes, this is not sufficient for understanding the complex sugar metabolism during acquisition of desiccation tolerance. Proteomics defines the entire protein complement in a cell, tissue, or organism [58]. As a major -omics technology, proteomics facilitates a system-based approach to desiccation tolerance. In this section, we will review sugar metabolism in desiccation tolerant plants studied by proteomics approaches.

Early in 1986, Harten and Eickmeier compared the activities of 10 enzymes in dried and hydrated *S. lepidophylla* fronds. Dried tissues retained an average of 74% enzyme activity [59]. Photosynthetic carbon assimilation enzymes, such as RuBP carboxylase and (NADPH)-triose-phosphate dehydrogenase retained the lowest level of activities, whereas the enzymes associated with respiration—e.g., malate dehydrogenase and enolase—retained the highest activity. Using the same plant species, Wang et al. (2010) studied the proteomes of *S. lepidophylla* at various RWCs upon dehydration [60]. The abundance of most proteins with respect to sugar metabolism fluctuates in response to different RWCs. For instance, the abundance of chloroplast ribose-5-phosphate isomerase, phosphoglycerate kinase, and aldose decreased one day after dehydration and recovered three days after dehydration; in contrast, the abundance of 6-phosphogluconate dehydrogenase increased at 49% RWC and recovered at 19% RWC.

The summary of available proteomic studies on resurrection plants, including *S. lepidophylla* [60], *S. stapfianus* [61], *Physcomitrella patens* [62], *B. hygrometrica* [63], and *X. viscosa* [64,65], reveals three main observations concerning the sugar metabolism relevant for adaption to dehydration.

The first is that the levels of the large subunit of ribulose-1,5-bisphosphate carboxylase/oxygenase (RuBisCO) decline sharply and the levels of some glycolytic enzymes, e.g., GAPDH, increase during drying, suggesting downregulation of photosynthesis and the requirement of both NAD(P)H and ATP. Secondly, enzymes participating in the Calvin cycle are still playing important roles in resurrection plants during dehydration, though the net photosynthetic rate is decreasing. For example, the abundance of chloroplastic phosphoglycerate kinase, glyceraldehyde-3-phosphate dehydrogenase, chloroplastic aldolase, sedoheptulose 1,7-bisphosphatase, and phosphoribulose kinase in *S. stapfianus* increased at 30% RWC [61]. The third characteristic is that enzymes with multiple functions are modulated in different ways in response to dehydration. Transketolase has been one of the most frequently identified proteins in proteomes of resurrection plants. It occupies a pivotal place in metabolic regulation, as it provides a link between the glycolytic pathway and the pentose phosphate pathway. In *S. lepidophylla* [60], the abundance of some transketolase proteins decreased while some others increased three days after dehydration. Similar observations were made in *P. patens* [62]: the abundance of transketolase proteins was both reduced and increased by dehydration. There are close connections between photosynthesis, glycolysis, the pentose phosphate pathway (PPP), and gluconeogenesis in plants. Some reactions of the non-oxidative PPP and Calvin cycle (photosynthesis) and Entner-Doudoroff pathways (glycolysis) overlap. By sharing intermediate metabolites (e.g., fructose-6-phosphate and glyceraldehyde-3-phosphate), the PPP is connected with glycolysis. The light-independent reactions of carbon fixation in the Calvin cycle share enzymes and reactions with the pentose phosphate pathway (e.g., the transformation of ribose-5-phosphate into ribulose-5-phosphate catalyzed by ribose-5-phosphate isomerase) and glycolysis (e.g., the transformation between glyceraldehyde-3-phosphate and dihydroxyacetonephosphate catalyzed by triosephosphate isomerase) [66]. Thus, only further identification of the cellular localization and properties of enzymes with multiple roles can help to understand the contribution of these enzymes to desiccation tolerance. The studies on transketolase of *C. plantagineum* from Bernacchia et al. (1995), Willige et al. (2009), and Zhang et al. (2016) provide an example for this proposal: three isoforms of transketolase (tkt3, tkt7, and tkt10) have been characterized on the molecular level in *C. plantagineum*; tkt3 is localized in chloroplasts while tkt7 and tkt10 are localized in the cytoplasm; tkt3 is suggested to be involved in carbon reactions in photosynthesis and the pentose phosphate pathway, while tkt7 and tkt10 are involved in the accumulation of octulose in *C. plantagineum* [67,68,69].

## 6. The Relationships among Sugar Accumulation, Energy Metabolism, and Other Metabolites

When sugars are accumulating during dehydration, various other changes occur simultaneously in resurrection plants, including cessation of photosynthesis, rearrangement of cellular structures, cell membrane remodeling, anthocyanin synthesis, ROS scavenging, and transition of protein metabolism from an active state to a stationary state. To respond to these changes, cellular energy will be redistributed and the corresponding metabolic pathways are rearranged.

Carbohydrates as products of photosynthesis act as resource and storage of energy. The levels of carbohydrates reflect to some extent the preparation of plants for desiccation stress. The total carbohydrate content changes during dehydration. For instance, total carbohydrate content both in leaves and roots of *C. plantagineum* decreases after dehydration [25,70], while it increases during dehydration in leaves of *X. viscosa* [23], *B. hygroscopica* [24], and *M. flabellifolia* [27]. To explain this observation, the dynamics of carbon assimilation/dissimilation and the transformation between carbohydrates and other organic compounds during dehydration need to be analyzed. Photosynthesis is vital for plant growth and development. The response of desiccation tolerant plants to dehydration in terms of photosynthesis has been reviewed by Dinakar et al. (2012) [71]. Although net photosynthesis rate in resurrection plants declines during dehydration, some carbon is still assimilated before the rate reaches zero. In this context, the transformation between carbohydrate and other organic compounds determines the total carbohydrate level.

Starch is observed in several resurrection plants, e.g., *C. wilmsii*, *C. plantagineum*, and *X. viscosa* [23,72,73]. Generally, starch is seen as carbohydrate storage similar in desiccation sensitive plants and it is degraded during dehydration [28,74]. Octulose in Craterostigma species may have a similar role as starch in providing carbon resources for sucrose or RFOs synthesis [26,70]. Zhang et al. (2016) demonstrated that *C. plantagineum* tkt7 and tkt10 catalyze the synthesis of octulose-8-phosphate using glucose-6-phosphate and fructose-6-phosphate as substrates [69]. Besides starch degradation, dehydration also induces alterations of the polysaccharide contents and structure in the cell wall of resurrection plants [75]. In *C. wilmsii*, the glucose content in the hemicellulose fraction was lower in dry plants than in hydrated plants and xyloglucan from the cell wall of dry leaves was relatively more frequently substituted by galactose than in cell walls of hydrated plants [75]. The change in carbohydrate composition of cell walls is also a part of carbohydrate metabolism during dehydration.

Metabolism of lipids, amino acids/proteins and nucleic acids are closely related to sugar metabolism. Besides sugars, many resurrection plant species also accumulate amino acids [76] and in particular proline [77]. For instance, in *S. stapfianus*, amino acids such as asparagine, arginine, glutamate, glutamine, and the amino acid precursor quinate accumulate during desiccation [78]. Thus, a competition for carbon skeletons and energy exists between sucrose synthesis and amino acid synthesis. Whittaker et al. (2007) examined this competition by analyzing SPS activity and other metabolic checkpoints involved in the co-ordination of carbon partitioning during dehydration in *S. stapfianus* [74]. They proposed that during the initial phase of dehydration (from 100% to 70% RWC), photosynthesis and starch breakdown sustain the simultaneous increase of hexose sugars, sucrose, and amino acids; at the second phase of dehydration, the activity of SPS facilitates rapid accumulation of sucrose, whereas the increase in amino acids in this phase is probably due to the breakdown of proteins and subsequent amino acid transformations. Quartacci et al. (1997) found that dehydration resulted in a reduction of lipids in leaves of *S. stapfianus* [79]. Similarly, in *Ramonda serbica*, dehydrated leaves suffered a reduction of about 75% in their plasma membrane lipid content compared with well-watered leaves [80]. In contrast, in *C. plantagineum* the total lipid content remains constant while the lipid composition undergoes major changes during desiccation [81]. This indicates that the transformation from lipids to carbohydrates is possible during dehydration. Carbohydrate metabolism and nucleotide synthesis can be linked through the pentose phosphate pathway that plays a crucial role in the supply of ribose-5-phosphate which is the molecular backbone of nucleic acids [66].

**Figure 1:**
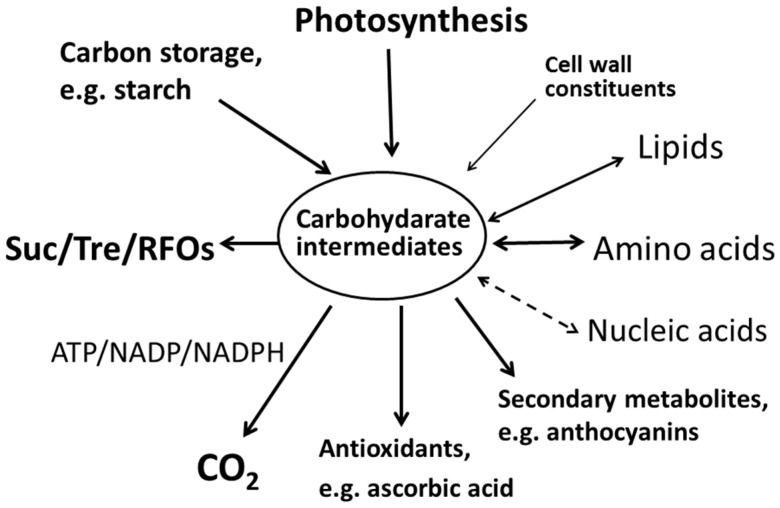
The cellular activities related to sugar accumulation in plant desiccation tolerance. The arrows represent the direction of reaction/transformation and the thickness of the line represents the strength of reaction/transformation. The dotted line indicates the transformation has not been demonstrated experimentally to date. “Suc”, “Tre”, and “RFOs” indicate sucrose, trehalose, and raffinose family oligosaccharides, respectively.

During dehydration the production of ROS is increased in plant tissues. Generation of ROS is an indispensable process for all aerobic organisms during dehydration. To prevent damage associated with oxidative stress, resurrection plants have evolved various protective mechanisms [82]. Many resurrection plants, such as *L. brevidens* [69] or *M. flabellifolia* [68] will accumulate anthocyanins that have a basic structure composed of carbon and act as photoprotective light screens in vegetative tissues [83]. To respond to ROS produced by dehydration stress, plants induce antioxidant defense systems. Sgherri et al. (1994) showed that after dehydration, H_2_O_2_ production decreased whereas total ascorbate and glutathione levels in *B. hygroscopica* increased two and 50 times, respectively [84]. The proteomic studies of *P. patens* [62], *S. lepidophylla* [60], and *X. viscosa* [64] all found the existence of GDP-mannose 3,5-epimerase that catalyses the conversion of GDP-mannose to GDP-L-galactose or GDP-L-gulose, and represents the first step in the de novo synthesis of ascorbate [85]. This suggests that the defense system of plants to oxidative stress is closely linked to carbohydrate metabolism. Thus the ROS defense system competes for carbon compounds. In addition, there are other metabolic pathways which are linked with carbohydrates, e.g., phenolics could also participate in a detoxification system of hydrogen peroxide during the dehydration/rehydration cycle [86]. The scheme in Figure 1 illustrates the relationship between sugar accumulation and other cellular biochemical activities.

## 7. Unanswered Questions

Studies on sugar accumulation and the performance of key enzymes in sugar metabolism during dehydration stress are not sufficient to fully understand the regulation of sugar metabolism and the redistribution of energy in resurrection plants. So some open questions are proposed to further elucidate sugar metabolism in the context of plant adaptation to dehydration stress.

Firstly, the vital regulating role of sugars in gene expression and energy signaling has never been researched. In recent years, several regulatory circuits that link carbon nutrient status to plant growth and development have been revealed, including the glucose sensor (hexokinase, HXK), the trehalose-6-phosphate signal, the Target of Rapamycin (TOR) kinase pathway, and the growth inhibitory function of the SNF1-related Protein Kinase1 (SnRK1) as well as the C/S1 bZIP transcription factor network [12]. These pathways may interact closely with desiccation tolerance, but the interaction has not been investigated. Most resurrection plants accumulate large amounts of sucrose (or trehalose for some species). The relationship between sucrose/trehalose and their regulating function through these regulatory systems should be addressed.

Secondly, there is a diversity of sugar metabolism (e.g., sucrose and RFOs) across different resurrection plant species, this raises the questions: which role does each sugar play in desiccation tolerance and whether their functions are different? As efficient transformation protocols are lacking for resurrection plants, the role of sugars in desiccation tolerance cannot be revealed by mutational approaches. In contrast, some novel findings on sugars emerged with regards to the desiccation tolerance of *Saccharomyces cerevisiae* using mutants. Tapia et al. (2015) demonstrated that increasing intracellular trehalose is sufficient to confer desiccation tolerance to *Saccharomyces cerevisiae* and that a chemical property of trehalose is directly responsible for desiccation tolerance [87]. In addition, Petitjean et al. (2015) identified the Tps1 protein (trehalose-6-phosphate synthase) as a key player for yeast survival in response to temperature, oxidative, and desiccation stress [88]. It should be noted that the overexpression of genes involved in the synthesis of a variety of sugars and sugar alcohols (e.g., trehalose and galactinol) resulted in increased drought tolerance in experimental model plants (e.g., Arabidopsis or rice) [89,90].

Lastly, except common sugars, some sugar derivatives (e.g., sugar acid and sugar alcohol) also accumulate during dehydration. Their physiological functions need to be investigated. For example, the level of galacturonic acid, arabitol, and mannitol increase about two times in dehydrated *S. stapfianus* compared to hydrated plants [78]. The reason for the increase and their respective roles have not been investigated.

In summary, since understanding the biochemical role of sugars in vegetative desiccation tolerance is still at an early stage, more efforts should be made to study the enzymes catalyzing sugar metabolism. To date, studies reveal that sugar accumulation is important for desiccation tolerance acquisition, but no proof exists that sugar accumulation is indispensable in resurrection plants because tools for direct genetic modifications are not suited or presently lacking for resurrection plants. Proteomic studies have revealed that the transition of sugar metabolism during dehydration is complicated. For instance, the enzymes in the Calvin cycle are also functioning when glycolysis is generally thought to supply energy and reducing power to cells during dehydration. The overlaps of various metabolic pathways form a network, thus it is difficult to evaluate the impact of individual enzymes. The sugar metabolism also competes for carbon resources from other carbon biochemical activities. Sugar metabolism has a central role and is closely linked with other pathways. Sugar sensing and signaling in desiccation tolerance has not been investigated. So far, the systematic recognition of enzymes regarding sugar metabolism is not sufficient because of the drawbacks of proteomic technology [91] and the lack of genome sequence information of resurrection plants [92]. However, increasing metabolomic studies are providing missing links and the published genome of novel resurrection plants, *P. patens* [93], *B. hygrometrica* [94], and *Oropetium thomaeum* [95] will accelerate our understanding of sugar metabolism in adaption to desiccation tolerance.

## Figures and Tables

**Table 1:** Sugar composition in representative resurrection plants.

Species *	Sugar Composition in Percentage		References
	Hydrated	Desiccated	
*Porella platyphylla* ^A^	Glucose 0.9%	Glucose 2.6%	(Marschall et al., 1998) [19]
	Fructose 0.7%	Fructose 2.3%	
	Sucrose 39.4%	Sucrose 44.6%	
	Fructan 59.0%	Fructan 50.6%	
*Selaginella lepidophylla* ^B^	Fructose 0.1%	Fructose 0%	(Adams et al., 1990) [20]
	Glucose 3.1%	Glucose 0.2%	
	Mannitol 0%	Mannitol 0.1%	
	myo-Inostitol 0.1%	myo-Inostitol 0.03%	
	Sucrose 6.9%	Sucrose 23.1%	
	Trehalose 89.8%	Trehalose 75.6%	
	Trisaccharides 0.1%	Trisaccharides 0.2%	
*Eragrostis nindensis* ^C^	Sucrose 77.2%	Sucrose 68.6%	(Ghasempour et al., 1998) [21]
	Glucose 13.0%	Glucose 6.5%	
	Fructose 4.5%	Fructose 9.0%	
	Trehalose 5.4	Trehalose 2.2%	
	Raffinose 0.0%	Raffinose 8.0 %	
	Stachyose 0.0%	Stachyose 5.7%	
*Sporobolus stapfianus* ^C^	Sucrose 83.6%	Sucrose 99.7%	(Whittaker et al., 2001) [22]
	Glucose 8.2%	Glucose 0.1%	
	Fructose 8.2%	Fructose 0.1%	
*Xerophyta viscosa* ^C^	Fructose 11.1%	Fructose 2.2%	(Peters et al., 2007) [23]
	Glucose 16.6%	Glucose 2.8%	
	Sucrose 13.8%	Sucrose 39.8%	
	Galactinol 31.0%	Galactinol 0.0%	
	myo-Inositol 4.4%	myo-Inositol 0.5%	
	Raffinose 15.2%	Raffinose 29.3%	
	Stachyose 7.5%	Stachyose 18.1%	
	Verbascose 0.4%	Verbascose 7.4%	
*Boea hygroscopica* ^D^	Fructose 7.0%	Fructose 1.9%	(Albini et al., 1999) [24]
	Glucose 9.2%	Glucose 3.0%	
	Alditols 1.8%	Alditols 0 %	
	myo-Inositol 2.3%	myo-Inositol 0%	
	Sucrose 13.4%	Sucrose 91.0%	
	Cellobiose 0.9%	Cellobiose 0%	
	Gentiobiose 0.9%	Gentiobiose 0%	
	Galactinol 21.1%	Galactinol 1.0%	
	Raffinose l8.l %	Raffinose 2.5%	
	Melezitose 1.4%	Melezitose 0%	
	Maltotriose 0.9%	Maltotriose 0%	
	Stachyose 10.0%	Stachyose (traces)	
	Pentasaccharide 10.1%	Pentasaccharide (traces)	
	Hexasaccharide 2.8%	Hexasaccharide 0%	
*Craterostigma plantagineum* ^D^	Glucose 1%	Glucose 3%	(Bianchi et al., 1991) [25]
	Fructose 2%	Fructose 2%	
	Sucrose 5%	Sucrose 90%	
	Octulose 89%	Octulose 4%	
	myo-Inositol 1%	myo-Inositol 1%	
*Craterostigma pumilum* ^D^	Octulose 94.2%	Octulose 10.3%	(Egert et al., 2015) [26]
	Sucrose 2.4%	Sucrose 80.3%	
	Galactinol 0.3%	Galactinol 2.1%	
	Raffinose 0.8%	Raffinose 1.5%	
	Stachyose 1.1%	Stachyose 2.6%	
	Verbascose 1.3%	Verbascose 3.1%	
*Myrothamnus flabellifolia* ^D^	Trehalose 30.7%	Trehalose 38.1%	(Moore et al., 2007) [27]
	Fructose 25.9%	Fructose 7.0%	
	Sucrose 20.6%	Sucrose 38.9%	
	Glucose 17.0%	Glucose 12.1%	
	Stachyose 5.6%	Stachyose 1.7%	
	Raffinose 0.2%	Raffinose 2.2%	
*Ramonda nathaliae* ^D^	Glucose 3.6%	Glucose 2.1%	(Müller et al., 1997) [28]
	Fructose 4.6%	Fructose 2.1%	
	myo-Inositol 1.0%	myo-Inositol 0.8%	
	Sucrose 40.3%	Sucrose 82.6%	
	Raffinose 34.3%	Raffinose 9.4%	
	Galactinol 16.1%	Galactinol 2.9%	

* According to the systematic classification, plants are grouped into four groups: ^A^ liverworts; ^B^ ferns; ^C^ monocot angiosperms, and ^D^ dicot angiosperms.
